# The oral manifestations of syphilitic disease: a case report

**DOI:** 10.1186/s13256-019-2171-z

**Published:** 2019-07-26

**Authors:** Kaitlyn L. Streight, Ronald M. Paranal, Daniel M. Musher

**Affiliations:** Baylor College of Medicine, 1 Baylor Plaza, Houston, TX 77030 USA

**Keywords:** Oral syphilis, Syphilis, Hard palate

## Abstract

**Background:**

Syphilis is a sexually transmitted bacterial infection of the spirochete, *Treponema pallidum*. While primary syphilis often involves genitalia, oral manifestations are observed in a subset of patients. These lesions are often associated with submandibular and cervical lymphadenopathy. This is a case report of a primary syphilitic lesion located on the hard palate of the oral cavity, with only a very few cases described previously.

**Case presentation:**

We describe a rare case of syphilis in a 59-year-old African American man presenting with subjective fevers, chills, marked submental lymphadenopathy, a diffuse skin rash, and an ulcer of the hard palate.

**Conclusions:**

This case report demonstrates the importance of maintaining a high index of suspicion for syphilitic infection when a patient presents with nonspecific symptoms, a diffuse rash, and an oral lesion.

## Background

Syphilis is a sexually transmitted bacterial infection of the spirochete, *Treponema pallidum*. Infection is divided in three stages: primary, secondary, and tertiary. Approximately 2 to 3 weeks after inoculation of the organism, a primary lesion erupts as a painless papule that later ulcerates to form a chancre [1]. Although chancres are typically found on the genitalia, oral lesions are observed in a subset of patients who engage in oral sex. These lesions appear as painless indurated ulcers on the tongue, gingiva, soft palate, or lips, associated with lymphadenopathy affecting the submandibular and cervical regions [2]. The eruption of a painless macular rash affecting the palms and soles indicates progression to the second stage of the infection and occurs approximately 4 to 10 weeks after the emergence of the primary chancre. We describe a rare case of syphilis presenting with subjective fevers, chills, marked submental lymphadenopathy, a diffuse skin rash, and an ulcer of the hard palate. This is a rare report of a primary syphilitic lesion located on the hard palate of the oral cavity, with only a very few cases described previously [3–5].

## Case presentation

A 59-year-old African American man presented to our emergency department with a 1-month history of progressive submental swelling, subjective fevers, and chills. A review of systems was positive for dysphagia, sore throat, and significant weight loss. He reported a history of genital herpes simplex virus (HSV) infection and explained that he developed a cluster of multiple small, painful blisters on his penis 2 weeks prior to presentation but denied any other new genital lesions. He also admitted to multiple sexual partners in the past but stated that he had been sexually active with only one female partner during the previous year.

**Fig. 1:**
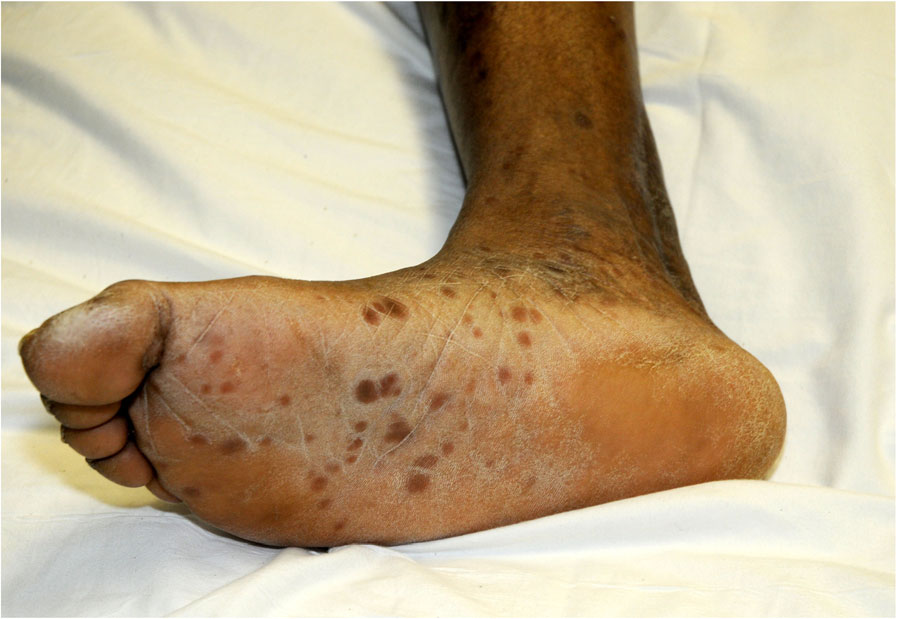
Diffuse erythematous maculopapular rash involving the soles of the feet

**Fig. 2:**
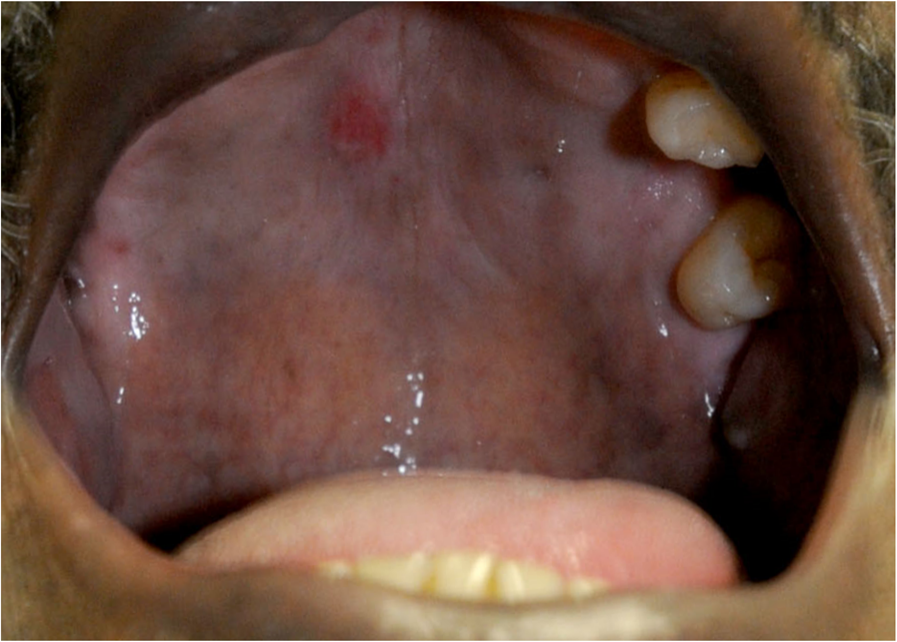
Primary syphilitic lesion located on the hard palate of the oral cavity

A physical examination revealed marked swelling and tenderness under his mandible and a diffuse erythematous maculopapular rash across his chest with scattered hyperpigmented macular lesions involving his palms and his lower extremities extending to the soles of his feet (Fig. 1). All lesions were nonpruritic and nontender, and he stated that he was unaware of the rash. Further examination revealed a 1 cm × 1 cm nontender ulcerative lesion on the hard palate of his oral cavity (Fig. 2). He was also unaware of this lesion on his hard palate. A genital examination revealed no lesions.

A computed tomography (CT) scan of his head and neck with contrast revealed marked lymphadenopathy from the submental region to his mid-neck with evidence of inflammatory changes and a partially necrotic left submental node.

His white blood cell (WBC) count was 12,500 with a neutrophilic predominance. Rapid plasma reagin (RPR) was reactive with a titer of 1:512. A Microhemagglutination Assay for *Treponema pallidum* (MHA-TP) was positive. Antigen and antibody tests for human immunodeficiency virus (HIV) infection were negative. Prior to the confirmed diagnosis of syphilis, a core biopsy of a submental lymph node was performed and revealed inflammatory changes with no evidence of malignancy. His cerebrospinal fluid was entirely normal and Venereal Disease Research Laboratory (VDRL) was negative. He received a single dose of 2.4 million units benzathine penicillin G intramuscularly and experienced marked improvement of his malaise and sore throat over the course of the next week. Four months after his initial presentation, repeat RPR titers were 1:8, and he reported complete resolution of his rash, lymphadenopathy, and dysphagia.

## Discussion

Syphilis is classified into three stages: primary, secondary, and tertiary. While these stages usually occur in sequence, they may overlap, as seen in our patient. The lesion of primary syphilis is described as a painless papule that emerges at the site of inoculation approximately 2 to 3 weeks after infection and later ulcerates to form a chancre [1]. While primary syphilis typically involves genitalia, oral manifestations are observed in approximately 4–12% of patients, reflecting sexual practices [2]. These lesions are often described as painless indurated ulcers commonly involving the tongue, gingiva, soft palate, and lips that typically last between 3 and 7 weeks [2]. Our patient presented with an ulcerated chancre on his hard palate, probably secondary to engagement in oral-genital sex with his partner. Other risk factors associated with the development of oral lesions include oral-anal sex and kissing [3]. Primary chancres on the hard palate are very rare and have only been reported in a few cases previously [3–5].

As the chancre develops, treponemes disseminate widely throughout the body. Disseminated lesions indicate progression to the second stage of infection and appear 4 to 10 weeks after the chancre is first seen, when a painless macular rash erupts over the trunk and extremities that extends to the palms and soles in a manner similar to our patient’s presentation (Fig. 1). As demonstrated by our case, many patients with secondary syphilis present with nonspecific symptoms such as fever, sore throat, weight loss, and lymphadenopathy [1]. Oral manifestations can also be seen during this stage and typically involve the soft palate and pillars, tongue, and vestibular mucosa [2]. Although oral manifestations of syphilis are relatively common, the lips serve as the most common site of oral lesions whereas lesions of the hard palate or labial commissure are very rarely reported and have previously occurred in the setting of multiple lesions [4, 6]. This case report is one of only a few reports of a primary syphilitic chancre on the hard palate of the oral cavity (Fig. 2), and our patient’s diffuse macular rash further indicated an overlapping progression to the second stage of infection (Fig. 1).

Our patient also presented with prominent submental lymphadenopathy characteristic of the regional lymphadenopathy seen in syphilitic disease. In approximately 80% of cases, syphilitic chancres are accompanied by painless regional lymphadenopathy, typically occurring 7 to 10 days after the appearance of the chancre [2]. While syphilis involving the genital region causes inguinal lymphadenopathy, lymphadenopathy can involve the cervical region in patients with oral syphilitic disease, as demonstrated by this case. In Chapel’s study of 105 patients with secondary syphilis, lymphadenopathy was present in the inguinal region of 79 patients, axillae of 40 patients, posterior cervical triangles of 29 patients, anterior cervical triangles of 24 patients, epitrochlear region of 18 patients, femoral region of 19 patients, and supraclavicular areas of 4 patients [7]. Characteristic pathology involves granulomas with epithelioid histiocytes, few multinucleated giant cells, and occasional necrosis, as seen in our patient [8].

## Conclusion

Oral syphilitic infection must be considered when a patient presents with oral lesions and dermatologic manifestations of secondary disease, even in the absence of genital ulcerations.

## Data Availability

Not applicable.

## Abbreviations

CT: Computed tomography
HIV: Human immunodeficiency virus
HSV: Herpes simplex virus
MHA-TP: Microhemagglutination Assay for *Treponema pallidum*
RPR: Rapid plasma reagin
VDRL: Venereal Disease Research Laboratory
WBC: White blood cell
